# Learning following Brain Injury: Neural Plasticity Markers

**DOI:** 10.1155/2019/4838159

**Published:** 2019-09-02

**Authors:** Leeanne Carey, Michael Nilsson, Lara Boyd

**Affiliations:** ^1^Occupational Therapy, School of Allied Health, Human Services and Sport, College of Science, Health and Engineering, La Trobe University, Bundoora, VIC 3086, Australia; ^2^Neurorehabilitation and Recovery, Stroke, Florey Institute of Neuroscience and Mental Health, Heidelberg VIC 3084, Australia; ^3^Faculty of Health and Medicine and Centre for Rehab Innovations, The University of Newcastle, Callaghan NSW 2308, Australia; ^4^LKC School of Medicine, Nanyang Technological University (NTU), Singapore 308232; ^5^Djavad Mowafaghian Centre for Brain Health, Faculty of Medicine, University of British Columbia, Vancouver, BC, Canada V6T 1Z3

Following a brain injury, individuals need to relearn lost skills, such as grasping, and learn new strategies to achieve goal-directed actions in daily activities. Yet the ability to learn and adapt is often disrupted. In this special issue, we called for new insights into the brain networks that support learning after brain injury, biomarkers of learning, and factors that may impact learning following brain injury.

In their review, L. Carey et al. developed and applied a novel methodology to identify key themes and topics to advance our thinking in relation to learning following brain injury, by searching the concepts of neuroplasticity, stroke recovery, and learning and finding the intersection of topics that link them. Using machine learning and natural language-processing technologies, the authors identified 23 intersecting themes (topics) from over a quarter of a million publications, with a time-linked pattern emerging. An important and unique feature of this approach was the ability to not only identify what is *common* across these contributing bodies of knowledge but also identify *gaps* in available literature. For example, while transfer of learning has been extensively researched in the learning literature, it did not emerge in relation to stroke recovery, neural plasticity, or their intersection. This highlights a gap in the knowledge base that informs stroke rehabilitation and also provides a clear direction for future research and potential applications from the field of learning. Overall, findings from this review identified foundation literature that may be synthesised to advance a neuroscience informed approach to stroke rehabilitation.

To date, attention has largely been focused on motor learning after stroke. Building on this literature, F. Alnajjar et al. employed a computational approach to investigate the motor control system for adaptation in healthy individuals and in recovery poststroke. They aimed to determine whether neuromuscular control strategies are comparable between healthy individuals during their adaptation to an unfamiliar environment and stroke survivors during their recovery. Results revealed that computed muscle synergy characteristics changed both in healthy participants under unfamiliar environment conditions and in stroke survivors following motor recovery. The authors concluded that change in muscle synergies during recovery from moderate stroke most likely represents an adaptation of existing synergies, similar to what occurs in healthy individuals when neurons adapt to an unfamiliar environment. Further, a relationship between muscle synergies and energy consumption was found. Findings suggest that training leads to gradual adaptation to the new environment, with implications for energy consumption.

Knowledge of brain networks that support learning after brain injury is critical not only to advance knowledge of biomarkers of recovery facilitated by learning-based therapies but also to guide development of tailored interventions. K. Wadden et al. report on white matter biomarkers associated with motor change in individuals with stroke. Individual variability is identified as a key issue in the application and effectiveness of adjunctive therapies, such as continuous theta burst stimulation (cTBS, i.e., repetitive brain stimulation), when paired with skilled motor practice. The authors investigated the white matter microstructure of a motor learning network, named the constrained motor connectome (CMC), as well as the corticospinal tract (CST) of lesioned and nonlesioned hemispheres. Individuals categorised as responders vs. nonresponders, based on change in motor behaviour, showed significant differences in the microstructural properties in the CMC, but not in CST. These findings revealed a potential new biomarker for training-facilitated motor recovery that extends beyond the CST alone. The relationship between the complex white matter motor network and the responsiveness of individuals to cTBS paired with motor practice was highlighted.

P. Goodin et al. looked beyond sensorimotor networks and brain structure to whole brain functional regions that may be important in learning and recovery. Factors such as mood, common poststroke, are associated with poorer recovery and worse cognitive outcomes and negatively impact response to rehabilitation in acute and subacute phases of recovery. The authors therefore sought to investigate the relationship between level of depressive symptom score and intrinsic brain activity in varying brain regions in 63 stroke survivors at 3 months poststroke. They investigated changes in low-frequency fluctuations in brain signals associated with poststroke depressive symptoms, specifically whether interaction effects might be observed. Significant interaction effects were found, involving frontostriatal and cerebellar regions, including insula. Further investigation is recommended given the role of these regions in sensorimotor processing and learning.

Two papers advance our understanding of potential biological markers of brain plasticity and learning through animal models of brain injury. J. Houlton et al. investigated the involvement of brain-derived neurotrophic factors (BDNF) in improving learning in aged mice after stroke. They established an animal model of stroke that induced delayed impairment in spatial memory. Spatial performance and memory were trained and monitored using a touchscreen and visual pairwise discrimination task. The treatment group received a BDNF decoy, TrkB-Fc. Aged mice exhibited greater stroke-induced cognitive deficits relative to young controls but also significant improvement in learning, which was dampened in the presence of the BDNF decoy. As concluded by the authors, these findings suggest age-related differences in recovery of cognitive function, with potential reopening of a critical window for recovery that is being mediated by BDNF. The role of BDNF in improving learning in aged mice after stroke was revealed.

B. Pijet et al. presented data on the influence of matrix metalloproteinase-9 (MMP-9) on dendritic spine density and morphology in an animal model of traumatic brain injury (TBI). The injury caused a marked decrease in spine density as well as spine shrinkage in the cerebral cortex ipsilateral to the injury, when compared to sham animals and the contralateral side, both one day and one week after the insult. Decreased spine density was also observed in the dentate gyrus of the hippocampus. In mice lacking MMP-9, no effects of TBI on spine density and morphology were observed, further implying a role for MMP-9 in brain plasticity.

Through this special issue, we have sought to bring together a themed collection of new insights and pathways to the investigation of learning following brain injury, focusing on markers of neural plasticity. We thank the authors for their contributions and hope this issue serves to stimulate further research across multiple disciplines and fields of related research. Perhaps in a decade, a similar review to that conducted by L. Carey et al. may reveal a rich intersection of knowledge across the fields of neural plasticity, learning, and stroke recovery?

